# Sigmoid Volvulus Causing Closed-Loop Obstruction and Surgical Management in a 16-Year-Old Female: A Case Report and Literature Review

**DOI:** 10.7759/cureus.81396

**Published:** 2025-03-29

**Authors:** Ebrahim Matar, Farah Naser, Hatim Ahmed, Yusuf Yusuf

**Affiliations:** 1 Surgery, Antrim Area Hospital, Antrim, GBR; 2 General Medicine, Altnagelvin Hospital, Derry, GBR; 3 General and Colorectal Surgery, Antrim Area Hospital, Antrim, GBR; 4 Surgery Department, Harrogate District Hospital, Harrogate, GBR

**Keywords:** bowel obstruction, child and adolescent, hartman’s procedure, rare case of sigmoid volvulus, sigmoid volvulus

## Abstract

Sigmoid volvulus (SV) is a rare cause of bowel obstruction in children and has serious implications. In adolescents, diagnosis can be challenging due to nonspecific symptoms. Prompt diagnosis and intervention is critical to avoid complications such as sepsis or death. Treatment options for SV include non-operative and operative management, with immediate surgical intervention in those with ischaemia, perforation, or failed non-operative management. We present a 16-year-old female that presented to the emergency department twice with SV, complaining of vomiting and abdominal distention. An abdominal XR was conducted showing dilated loops of bowel. The diagnosis of SV was later confirmed by CT imaging. After failure of decompression with flexible sigmoidoscopy, the surgical team proceeded with open Hartmann's procedure. Intraoperatively, a long loop of ischaemic sigmoid colon was identified and twisted 360 degrees in an anti-clockwise direction. Surgical intervention via open Hartmann’s procedure was performed, and the patient had an uneventful recovery with ICU care and supportive management. Assessment of the specimen identified a congenital band, which seems to have led to SV.

## Introduction

Sigmoid volvulus (SV), although rare in adolescents, is a serious cause of intestinal obstruction that can lead to life-threatening complications if not promptly diagnosed and treated [1]. Volvulus is an acute condition that occurs as a results of twisting of the bowel, which can lead to ischemia or bowel obstruction, depending on the degree of twisting [1]. It is more commonly observed in older adults, seen particularly in males from eastern countries [1]. In the pediatric population, SV presents mostly in patients with chronic constipation or Hirschsprung's disease, but SV can also occur in individuals without these typical risk factors [2]. The condition results from the twist of the sigmoid colon around its mesenteric axis, leading to bowel ischemia, obstruction, and potential perforation [2].

We present this rare case of a 16-year-old Caucasian female, presenting to a UK hospital, with vomiting and abdominal distention over five days. Notably, there was no history of constipation or any preceding symptoms, leading to a misdiagnosis of gastritis. Given the patient's worsening condition, she represented to emergency department (ED) where surgical intervention became necessary after failure of endoscopic decompression. Hartmann’s procedure was ultimately deemed the most appropriate choice due to the severity of ischemia. Postoperatively, she was admitted to the intensive care unit (ICU) and had an unremarkable recovery.

## Case presentation

A 16-year-old female presented initially to the ED with six episodes of vomiting and abdominal distention over the last 24 hours. She denied any abdominal pain or diarrhea. On examination, her abdomen was soft and non-tender, but it was distended and hyper-resonant to percussion. She was tachycardic with a normal temperature, respiration, and blood pressure and a National Early Warning Score (NEWS) of 2. Initial lab work revealed mildly elevated white cell count (WCC) at 12.2 x 10^9^/L (normal range 4.5 to 11.0 x 10^9^/L) and a normal C-reactive protein (CRP) of 2.0 mg/dL (normal range 1-5 mg/dL). The patient was otherwise healthy with no significant past medical history. She had no previous abdominal surgeries or history of constipation. The patient was managed with anti-emetics and analgesia; after settling in the ED, she was discharged, with a diagnosis of gastritis.

**Figure 1:**
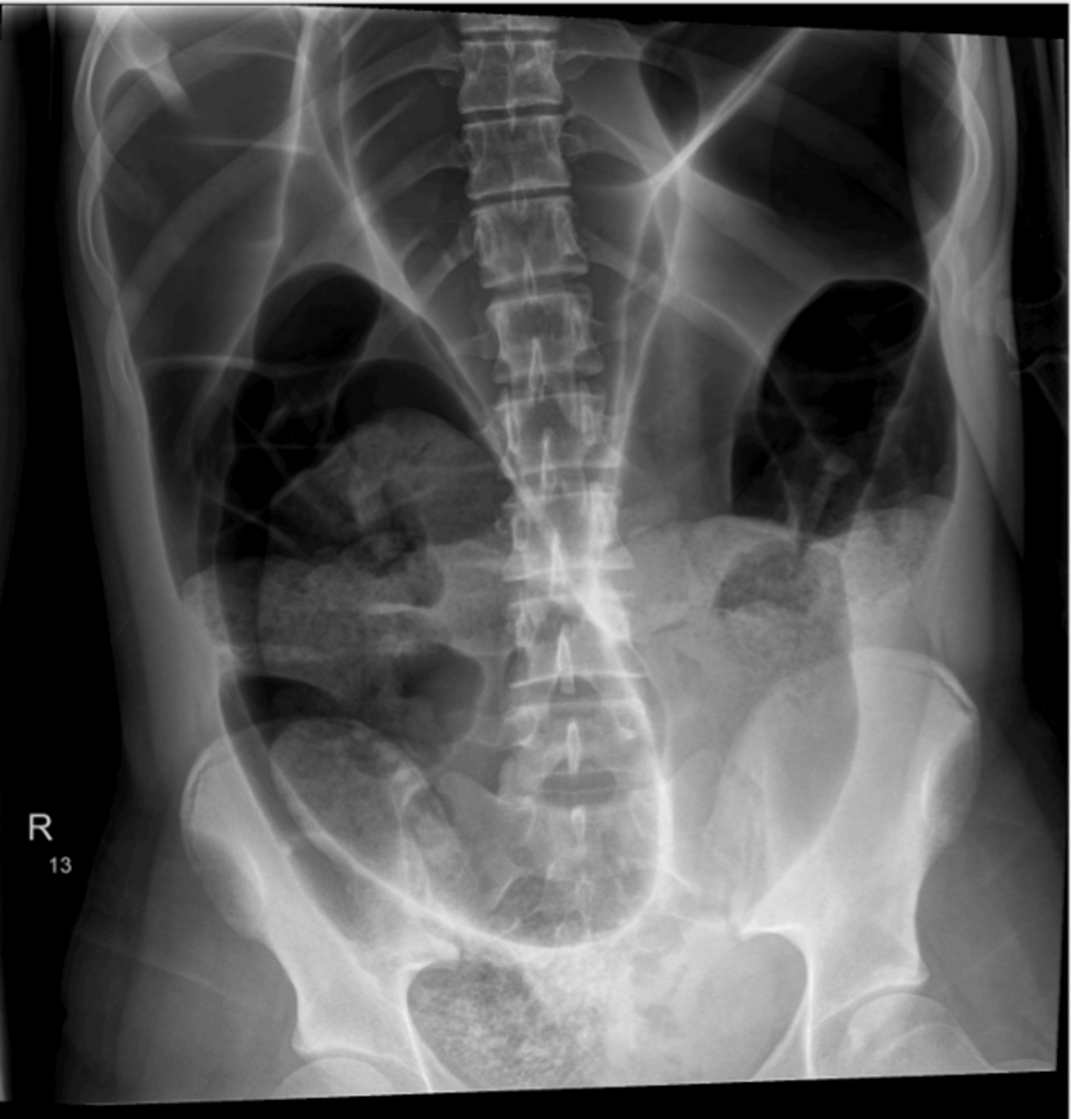
An abdominal radiograph showing a coffee bean sign

Two days later, she returned to the ED with worsening of her symptoms, including multiple episodes of vomiting (four times a day), abdominal tightness, and a four-day history of constipation with inability to pass flatus. On examination, she had a distended abdomen and generalized abdominal tenderness, tympanic abdomen, and reduced bowel sounds. She remained tachycardic with a normal temperature, respiration, and worsening of her NEWS at 3. Laboratory results showed significant abnormalities with further elevation of her WCC at 26.3 x 10^9^/L, CRP at 155 mg/dL, new acute kidney injury (AKI) of stage 1, and an elevated lactate at 4.8 mmol/L. Urine dipstick was negative, and a pregnancy test was also negative. Abdominal X-ray (AXR) revealed dilated loops of bowel, showing the coffee bean sign, a classical finding in SV (Figure 1).

**Figure 2:**
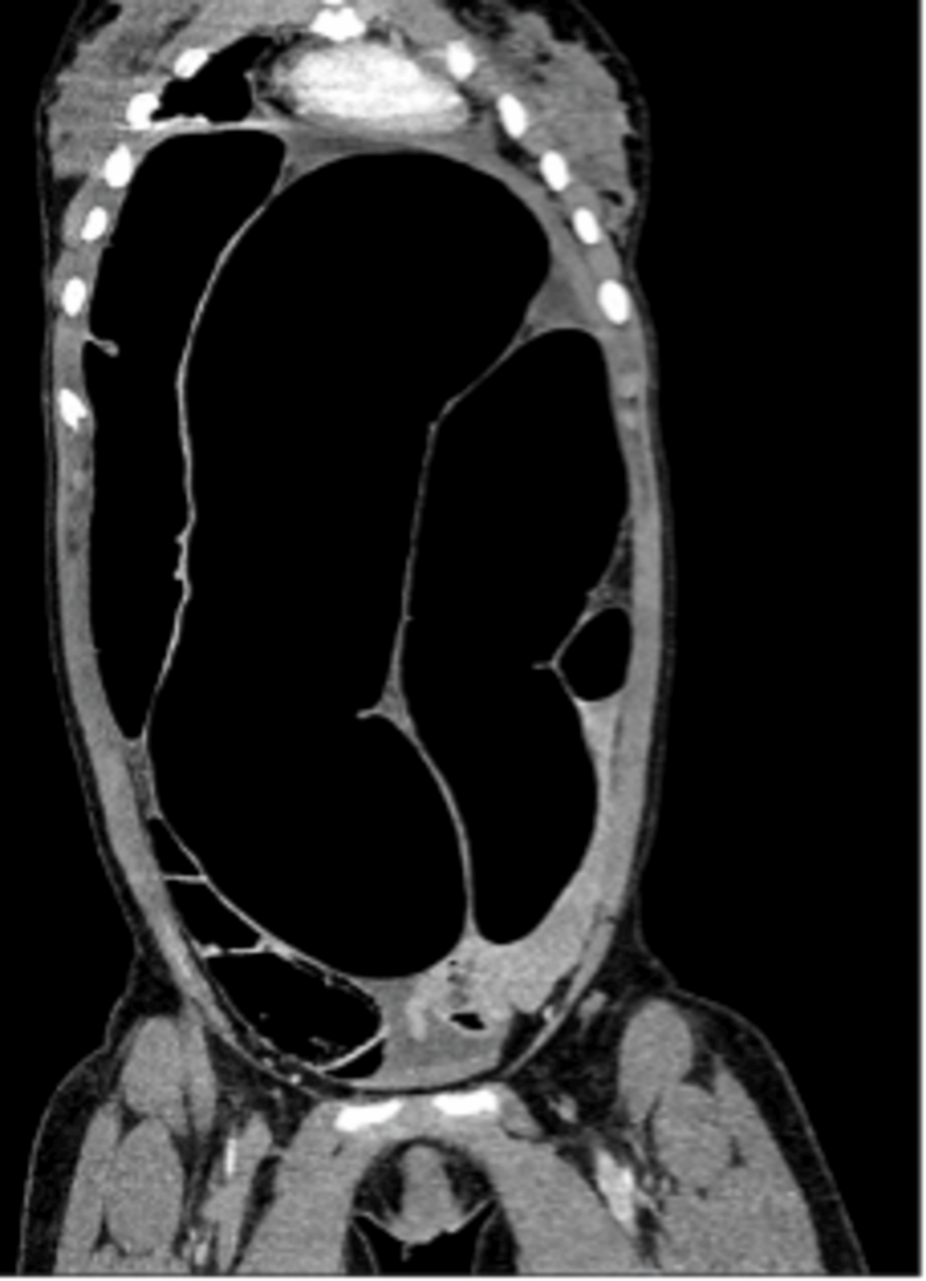
Contrast-enhanced computed tomography of the abdomen and pelvis (CT AP) showing the dilated loop of the large bowel

**Figure 3:**
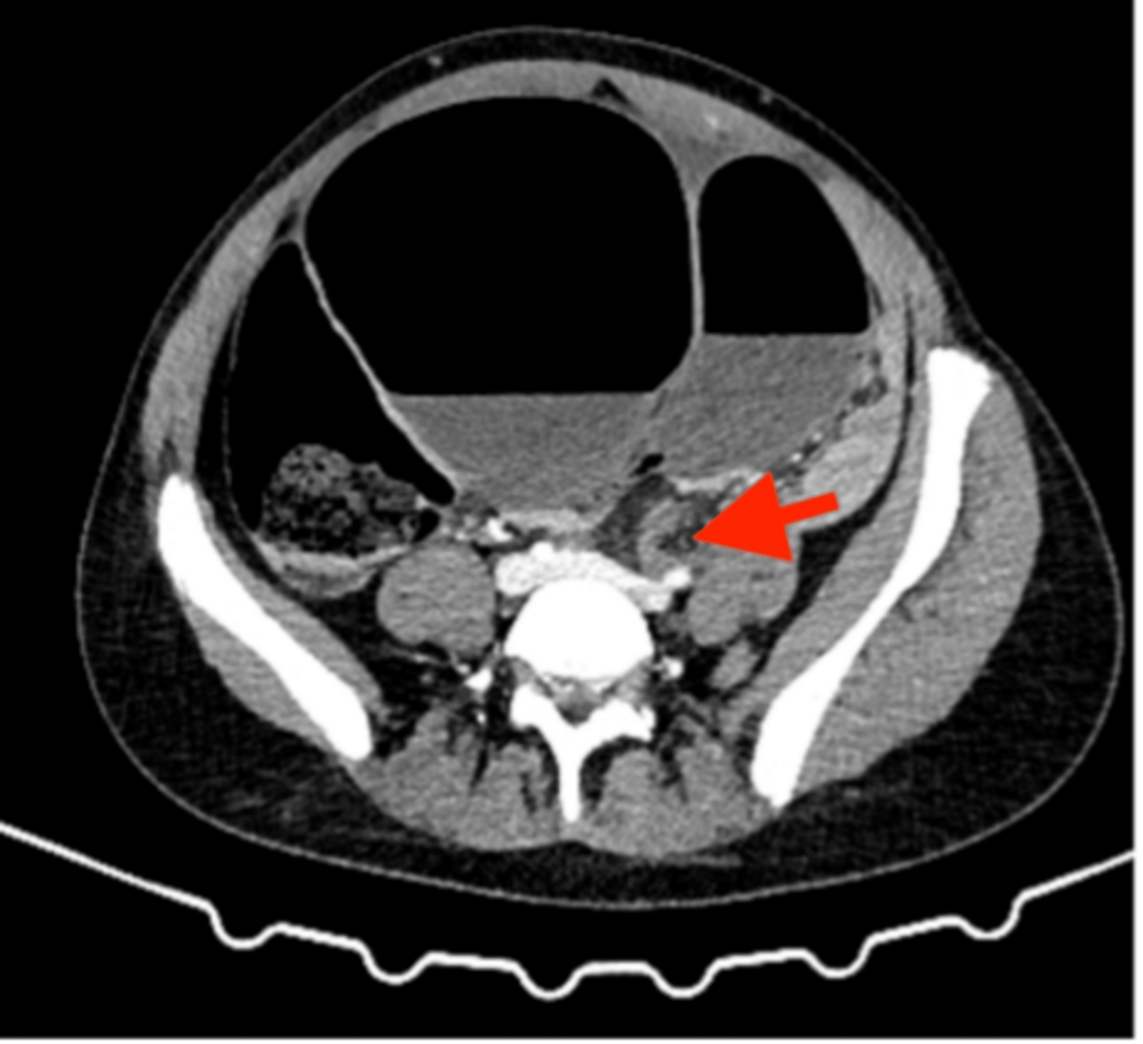
Abdominal computed tomography (CT) showing the swirling sign and transition point indicated by the red arrow

A contrast-enhanced computed tomography of the abdomen and pelvis (CT AP) revealed SV, causing a closed-loop obstruction (Figures 2, 3). CT AP also showed a coffee bean viscus of sigmoid colon demonstrating swirling and cartwheeling with a beak appearance of the transition point, which are indicative of SV. No evidence of perforation or bowel ischaemia on CT AP.

Management

The patient was kept nil by mouth (NBM) and started on fluid resuscitation and intravenous antibiotics; then, a urinary catheter was inserted. Given the clinical stability of the patient, it has been decided to attempt decompression via flexible sigmoidoscopy, which was successful. Flexible sigmoidoscopy showed SV with mild to moderate ischemia, sigmoid colon detorsion, and hemorrhagic fluid in the sigmoid colon.

During recovery, a couple of hours after endoscopic decompression, the patient clinically deteriorated and therefore was sent for another flexible sigmoidoscopy. During endoscopy, the sigmoid was visualized, which showed ongoing ischemia. Subsequently, the decision was made to proceed with a laparotomy for definitive surgical management.

**Figure 4:**
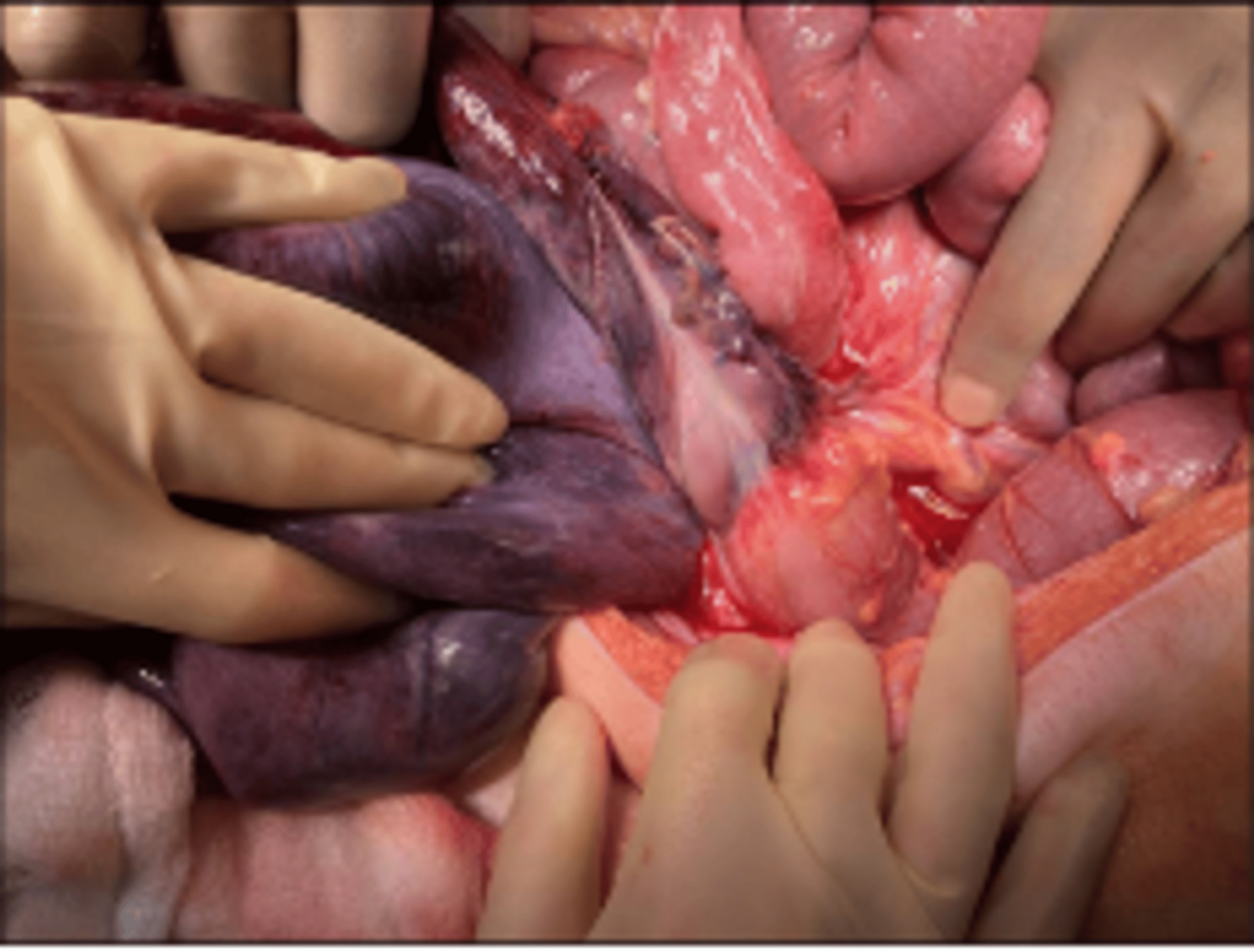
Sigmoid twisted anti-clockwise

**Figure 5:**
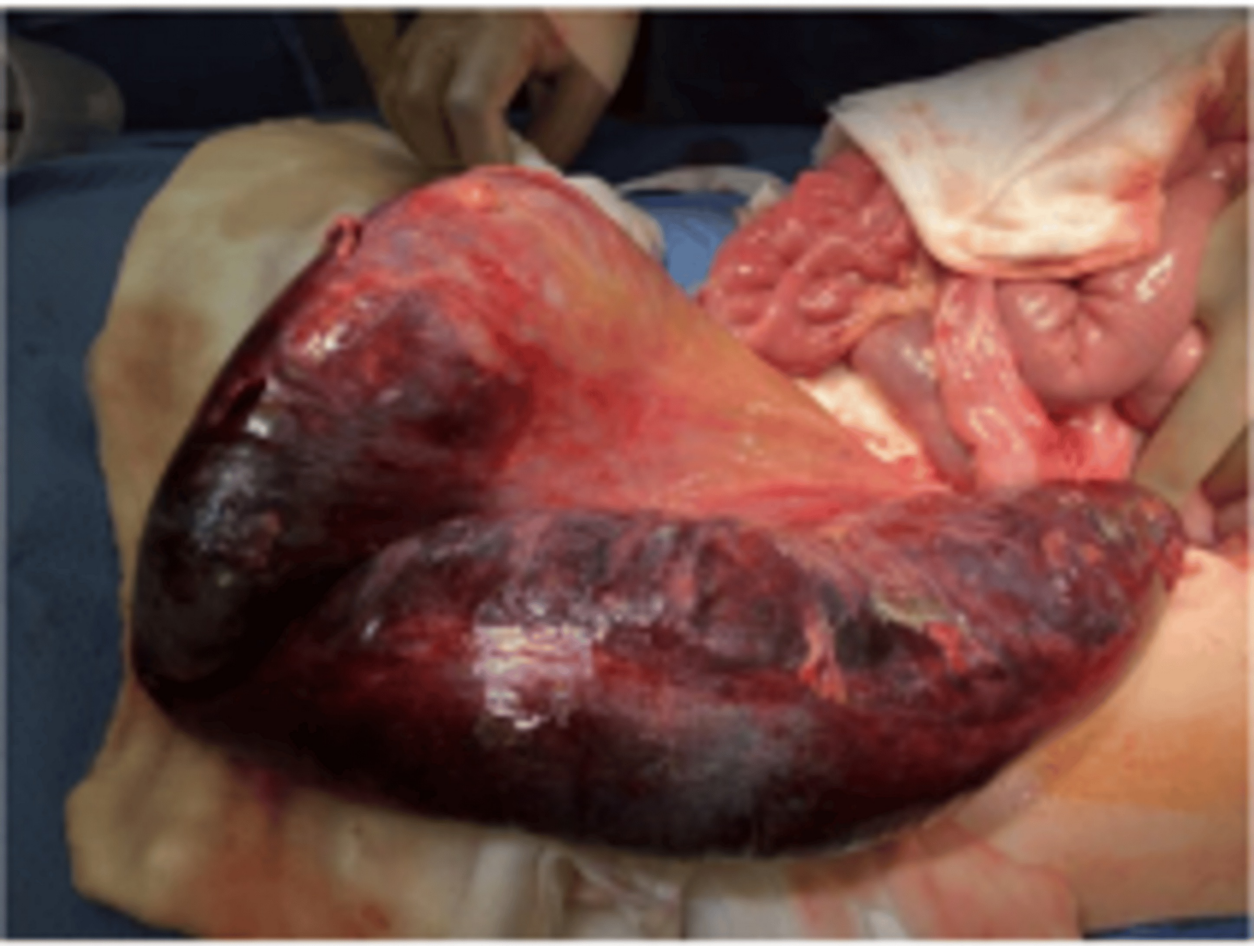
Ischemic sigmoid

During the laparotomy, a long loop of the ischemic sigmoid colon was found, twisted 360 degrees in an anti-clockwise direction (Figure 4). The ischemic segment extended from the distal sigmoid colon to the upper rectum (Figure 5). There was no evidence of bowel perforation. The transverse, ascending colon and the cecum were markedly dilated, with normal small bowel, indicating a closed loop large bowel obstruction.

A Hartmann’s procedure was performed, which included the resection of the ischemic segment of the sigmoid colon and the creation of an end colostomy. The patient tolerated the procedure well, and no immediate complications were noted. The specimen was sent for pathology assessment, which identified a congenital band, that seems to have been the precursor to SV.

Postoperatively, the patient was transferred to the ICU for close monitoring for three days. She was started on total parenteral nutrition (TPN) and had a nasogastric (NG) tube inserted for decompression. She showed steady improvement and was eventually transferred to the ward after stabilization.

## Discussion

SV is uncommon in adolescents, yet it carries significant risk, including perforation or ischemia, which if not identified promptly lead to sepsis and possibly death [3,4]. SV is the result of rotation of the sigmoid and is mesentery, which occurs due to a mobile sigmoid colon rotating around its mesentery [3,4]. This results in the loss of blood supply to the sigmoid [5]. Complication of SV depends on the degree of rotation, and obstruction occurs when the sigmoid is twisted more than 180 degrees, while ischemia occurs when the sigmoid is twisted more than 360 degrees [1].

There are a number of predispositions to SV in the pediatric population that has been reported in the literature inclusive of anatomical abnormalities in the mesentery, malfixation, Hirschsprung's disease, chronic constipation, and neurodevelopmental disorders [2-5].

In a study by Jonathan Hencke in 2023, 256 cases of SV were reported in the pediatric population between 1941 and 2023, with a male-to-female ratio of 2.3:1 [4]. Of the 69 adolescent cases, 42 had no pre-existing conditions that predisposed them to SV, while 14 had chronic constipation [4]. This highlights the fact that adolescents with SV may present with no clear risk factors, making diagnosis a challenge [4].

One of the major challenges in diagnosing SV in pediatric and adolescent patients lies in the nonspecific and vague symptoms [3,4]. Abdominal pain, vomiting, and constipation are the most reported symptoms, while others, such as inability to pass flatus and diarrhea, are less frequent [3,4,6]. Abdominal distention is the most common clinical finding; however, in some cases, an abdominal mass or peritonitis may be noted [4]. The presence of abdominal pain, constipation, and vomiting, associated with abdominal distention, should raise the suspicion of SV [4,6].

Diagnosing SV in children requires a thorough history and clinical examination; this can be aided by radiological imaging [1,3,5]. If a clinician suspects SV, the most appropriate next step would be an AXR or CT AP [3]. Imaging studies can be helpful, though they have limitations [6]. In stable patients, an abdominal X-ray is a useful initial investigation, which can reveal the classic “coffee bean" sign; however, they are only diagnostic in about 30% of cases [2,4,6,7]. The use of barium enema increases the possibility of diagnosis SV significantly and has an additional therapeutic effect of possibly decompressing SV [1,7].

If uncertainty persists or if there is suspicion of perforation or ischemia, a CT scan of the abdomen and pelvis may be more diagnostic [1,5,6]. CT findings, such as the "whirlpool" or "bird’s beak" signs, are considered diagnostic of SV and can also provide valuable insight into the cause of the obstruction and its associated complications, which represents the rotation of the mesentery [1,5,6].

Management

Since SV is rare in the paediatric population, there is no clear guidance on the recommended treatment [1,3,7]. It is however agreed that the primary goal is to reduce the volvulus and prevent its recurrence [1,4]. The treatment of SV can be divided into non-operative and operative approaches, depending on the severity and response to initial treatment [4,5].

The experts in the literature are in agreements that in the acute setting non-operative management is appropriate for stable patients without signs of perforation or ischemia [1,4,5]. In cases where non-operative reduction fails or if there is evidence of perforation or ischaemia, surgical intervention is indicated [1,4,5].

Techniques include rectal tube placement, endoscopic reduction via sigmoidoscopy, barium enema, and proctoscopy, all of which have a success rate ranging from 33% to 91%. However, even after successful reduction, elective sigmoid colectomy is recommended to reduce the high recurrence rate, which goes up to 35% [1,3,5].

Surgical procedures in the emergency setting have higher complication rates, which is the reason that if indicated, non-operative management is recommended initially [6]. Options for surgical treatment, depending on the case, include sigmoid colectomy, mesentery-sigmoidopexy, sigmoidectomy, Hartmann’s procedure, and detorsion with either resection (end colostomy) or primary anastomosis [4,5,6].

## Conclusions

SV, while rare in adolescents, is a critical condition that requires quick identification and management to prevent serious complications such as ischemia, perforation, and sepsis. Its nonspecific presentation and low incidence in younger patients make diagnosis particularly challenging, often leading to delays in treatment. High clinical suspicion and early imaging, including abdominal X-rays and CT scans, play an essential role in confirming the diagnosis. Management options vary from non-operative reduction to surgical interventions, with the choice largely depending on the patient’s condition and the success of initial treatments.

This case highlights the importance of considering SV in the differential diagnosis of adolescents presenting with symptoms of bowel obstruction. This case underscores the importance of maintaining a high level of suspicion for SV in padiatric patients presenting with signs of bowel obstruction, even in the absence of traditional risk factors such as chronic constipation or underlying gastrointestinal disorders. Although SV is more commonly seen in older adults, it can occur in children.
